# Multi-omics for biomarker approaches in the diagnostic evaluation and management of abdominal pain and irritable bowel syndrome: what lies ahead

**DOI:** 10.1080/19490976.2023.2195792

**Published:** 2023-04-03

**Authors:** Andrea Shin, Purna C. Kashyap

**Affiliations:** a Division of Gastroenterology and Hepatology, Department of Medicine, Indiana University School of Medicine, Indianapolis, IN, USA; b Clinical Enteric Neuroscience Translational and Epidemiological Research Program, Division of Gastroenterology and Hepatology, Mayo Clinic, Rochester, MN, USA

**Keywords:** Microbiome, microbiota, metabolome, genetic, epigenetic, transcriptome, proteome, functional gastrointestinal disorder, connectome, disorders of gut–brain interaction

## Abstract

Reliable biomarkers for common disorders of gut–brain interaction characterized by abdominal pain, including irritable bowel syndrome (IBS), are critically needed to enhance care and develop individualized therapies. The dynamic and heterogeneous nature of the pathophysiological mechanisms that underlie visceral hypersensitivity have challenged successful biomarker development. Consequently, effective therapies for pain in IBS are lacking. However, recent advances in modern omics technologies offer new opportunities to acquire deep biological insights into mechanisms of pain and nociception. Newer methods for large-scale data integration of complementary omics approaches have further expanded our ability to build a holistic understanding of complex biological networks and their co-contributions to abdominal pain. Here, we review the mechanisms of visceral hypersensitivity, focusing on IBS. We discuss candidate biomarkers for pain in IBS identified through single omics studies and summarize emerging multi-omics approaches for developing novel biomarkers that may transform clinical care for patients with IBS and abdominal pain.

## Introduction

Irritable bowel syndrome (IBS) and other disorders of gut–brain interaction (DGBI) are common, chronic conditions that affect tens of millions across the globe. It is estimated that 4–10% ^1^ of the worldwide population suffer from IBS, while 40% meet criteria for other related DGBI (previously known as functional gastrointestinal disorders). Despite the notion that IBS is largely regarded as a benign condition, the cost and impact of IBS on society, the economy, and patients are substantial. It is associated with increased healthcare utilization,^2,3^, higher healthcare spending,^4–6^ decreased productivity,^6–8^ and impaired quality of life.^5,9–12^ For these reasons, developing cost-effective approaches to enhance diagnosis and management of IBS and other disorders characterized by chronic abdominal pain remains a high healthcare priority.

Identifying and validating actionable biomarkers are key strategies for improving the established paradigms of diagnosing, evaluating, and treating IBS.^13^ While a number of candidate biomarkers targeting both central and peripheral mechanisms of IBS have emerged over the years, achieving major breakthroughs in IBS biomarker development is challenged by the complex interindividual heterogeneity and intraindividual temporal instability that is intrinsic to the pathophysiology of the disorder. Furthermore, identifying and validating biomarkers of visceral hypersensitivity or abdominal pain, a defining feature of IBS, remains particularly challenging. The mechanisms leading to visceral hypersensitivity in IBS are complex, multifactorial, and incompletely characterized. Biological tools that capture quantitative measurements of visceral pain and are valid across the spectrum from experimental animal models to humans are limited. The lack of rigorous surrogate endpoints for visceral pain has further hindered the translation of bench to bedside research in IBS, particularly in drug development, which continues to rely on endpoints defined by subjective patient-reported symptoms.^14^ However, the ability to confront existing challenges and develop successful biomarkers may be aided through newer ‘omics-based approaches: metataxonomic and metagenomic sequencing of the intestinal microbiome, targeted and untargeted metabolomics, genomics, epigenomics, transcriptomics, proteomics, and connectomics for study of brain networks as well as integrated ‘omics-based studies’. Meanwhile, other evolving platforms such as single-cell RNA sequencing and spatial transcriptomics may present additional opportunities for novel insights the near future.

In this review, we will cover key findings related to candidate IBS biomarkers, emphasizing those that are linked to visceral hypersensitivity and chronic abdominal pain. We will review the role and importance of biomarkers in IBS; describe the pathophysiological mechanisms underlying visceral pain in the context of IBS through a critical summary of animal and human studies; evaluate individual ‘omics-based biomarker research for pain in IBS that have been derived from both preclinical and human studies with an emphasis on recent clinical research; and highlight the potential applications of multi-omics based approaches for the future discovery of novel and translationally relevant biomarkers that may help us decipher pain in this common, yet debilitating disorder.

## The need for novel ibs biomarkers

The concept of IBS pathophysiology was originally considered in the 1950s and 1960s and IBS was initially believed to be psychiatric illness.^15^ Through the modern era, progress has been made with the recognition of other peripheral and central features that offer a more nuanced understanding of this complex disorder within a biopsychosocial model. These features include altered motility or transit, impaired intestinal permeability, changes in intestinal secretion, immune cell activation, visceral hypersensitivity, and perturbed neural signaling within the enteric nervous system and bidirectionally along the gut-brain axis (reviewed in reference^16^. Advancements in the conceptual understanding of IBS pathogenesis have generated new avenues for research and guided the development of multiple therapies such as non-absorbable antibiotics, intestinal secretagogues, 5-HT_3_ receptor antagonists, neuromodulators, and diet-based treatments that are now approved or are available for patients with IBS.

Despite the body of knowledge and new treatment modalities that have accumulated over recent decades, IBS diagnosis and management of continue to rely on symptom-centered strategies. While a positive symptom-based diagnosis with limited testing remains the recommended approach endorsed by major society guidelines,^17,18^ this approach is complemented by empiric treatments that are selected based on clinical history and symptom phenotype. However, symptom phenotypes cluster individuals together who may suffer from variable and distinct pathophysiologic processes. Therefore, it is not surprising that many patients and clinicians remain dissatisfied and that the cost and burden of IBS remain high. In one survey of over 3000 patients with IBS, only a quarter to a third of patients reported being “very satisfied” with the use of Food and Drug Administration-approved agents for their IBS symptoms.^19^ These observations show that although reliable clinical diagnoses can be achieved and a wide array of treatments now exist, there is a critical gap between establishing the diagnosis and delivering effective care leading to durable improvements.

Current guidelines^17,18,20^ advocate for the use of limited laboratory and diagnostic testing, which in some cases constitute the use of a few select biological markers or biomarkers. However, these biomarkers are primarily aimed at ruling out alternative diseases (e.g., celiac disease, giardiasis, inflammatory bowel disease) rather than positively identifying pathophysiological targets for IBS therapy. Moreover, available tests do not address the specific feature of abdominal pain in IBS, which is independently correlated with increased symptom severity,^21^ impaired quality of life,^22^ and is a primary predictor for important clinical outcomes such as healthcare utilization.^23^ There is a clear need for usable biological assessments that will support a positive diagnosis of IBS, identify treatable mechanisms that underlie hypersensitivity, allow monitoring of treatment response, and promote cost-effective delivery of precision care. Under ideal circumstances, good biomarkers^24,25^ should provide objective, accurate and precise, reproducible, and quantifiable measurements that correlate with patient outcomes and provide clinically meaningful information beyond that which can be ascertained from history alone. Cost, feasibility, as well as patient acceptance are important additional considerations. Useful biomarkers should not require excessively invasive methods that subject patients to a potential risk of harm; acceptability and safety are especially important in the quest for biomarker discovery in IBS, a disorder that is associated with a major burden on patients and the healthcare system but carries an overall benign prognosis.

## Mechanisms underlying chronic visceral pain

Visceral pain associated with IBS may arise from peripheral sensory signaling as well as central processing, sensitization, and modulation of nociceptive pathways.^26^ While many insights have been gathered through preclinical and clinical studies, the full complexity of visceral hypersensitivity and pain is incompletely understood. Numerous factors including genetic variations; interactions between the gastrointestinal microbiome and host immune system and/or enteric nervous system; and perturbations in molecular events and chemical mediators of visceral nociceptive signaling may all contribute to the peripheral pathogenesis of abdominal pain in IBS.^26–28^ Centrally mediated mechanisms of pain in IBS that independently influence or augment peripheral pathways may further be affected by psychological factors, emotional distress or mood, chronic stressors, and bidirectional microbiome-gut-brain communication.^26,29^ Thus, a number of peripherally- and centrally mediated mechanisms of visceral hypersensitivity or pain have been explored in both preclinical and clinical studies of IBS (Table 1).

**Table 1.** Summary of mechanisms for visceral hypersensitivity (VH) or abdominal pain (AP) in irritable bowel syndrome (IBS) studied in both preclinical and clinical research.

Mechanism	Findings from Animal Studies	Findings from Human Studies
Mast cell activation	Mast cell activation required for aluminum-induced hypersensitivity in rats^30^ Colonization of germ-free mice with high histamine-producing fecal microbiota result in visceral hyperalgesia and mast cell activation^31^ IgE production by B cells and plasma cells leads to colonic mast cell activation and food-induced VH in a post-infection (PI)-IBS mouse model^32^ Synthesis and release of prostaglandin E_2_ by colonic mast cells activates the EP2 receptor on sensory neurons to induce VH in rats^33^	Improvement in visceral pain with mast cell stabilizer, ketotifen, in IBS^34^ VH and AP improved with ebastine (histamine receptor 1 antagonist) in IBS; visceral pain mediated by sensitization of submucosal neurons by mast cell mediators including histamine^35^ ↑mast cell activation and ↑IgE+ mast cells in closer proximity to nerve fibers in IBS versus controls; AP severity inversely correlated with distance between IgE+ mast cells and nerve fibers^32^
Barrier dysfunction	↑colonic permeability to sodium fluorescein associated VH in PI-IBS mouse model^32^ Inhibition of myosin light chain kinase-mediated intestinal permeability improved VH in PI-IBS mouse model^36^ IL-6 decreased tight junction protein, occludin, expression in colonic epithelial cells and induced visceral hyperalgesia in rats^37^	Barrier dysfunction prevalent in IBS, more common in t IBS with diarrhea (IBS-D) or PI-IBS, and positively associated with symptoms including AP^38^
Ion Channels	Sensitization of TRP vanilloid 1 (TRPV1) mediates VH in rodents^39,40^ Blockade of voltage-gated calcium channels (Ca_V_2.2, Ca_V_2.3) inhibits colonic nociceptor excitability in mice^41^ VH in DSS-treated WT mice associated with higher colonic expression of Cav3.2 mRNA In TNBS-induced VH mouse model, administration of selective Na_V_1.1-activating toxin enhanced mechanosensory responses^42^	Colonic TRPM8 mRNA expression associated with AP scores in IBS^43^ Rectosigmoid TRPV1-immuno-reactive fibers associated with AP scores in IBS^44^ Small intestinal TRPV1 or TRPV3 expression correlated with AP IBS^45^ Levels of TRPV4 agonist correlated with increased AP scores in IBS^46^ Otiliuim bromide (block L-type calcium channels), reduced AP in patients with IBS90 Pregabalin (binds α2δ subunit on voltage-dependent calcium channels) reduced pain and increase sensory thresholds in IBS, particularly in IBS-D.91, 92 *SCN5A*-encoded Nav1.5 sodium channel mutations associated with increased symptoms including pain^47^
SCFA	Intracolonic acetate enhances sensitivity to colorectal distention in rats^40^ Butyrate mediates VH in rats via enteric glial cell-derived NGF^48^ Butyrate enemas induced concentration-dependent ↓ in thresholds to colorectal distention in rats^49^	Direct administration of butyrate enemas decrease pain scores in healthy volunteers^50^
Neuropeptides	Upregulation of substance P in colon of mice with VH^51^	Positive correlation between colonic mucosal substance P and abdominal pain in IBS^52^
Endocannabinoid system	Cannabinoid receptor, CB1 and CB2, agonists mitigate AP and VH in rodents [reviewed in^53^)	Supplementation with endocannabinoid-like dietary compounds improve AP severity in IBS^54^
Direct effects of intestinal microbiota	Hypersensitivity to colonic distension of IBS patients c transferred to rats through their fecal microbiota^55^ *Clostridium sensu stricto* 1 abundance ↑ in rats with VH verses controls; treatment with probiotic VSL#3 decreased VH^56^ Rifaximin alters bacterial composition in the ileum and prevents visceral hyperalgesia in rats^57^	Relative abundance of colonic mucosal *Prevotella-9* correlated with increased AP in IBS^58^ Highest level of AP positively correlated with three *Bacteroides*, *Ruminoccocus*, and an unknown *Barnesiellaceae*; AP negatively correlated with *Prevotella, Catenibacterium*, and genus from the Erysipelotrichaceae family^59^ ↓AP following fecal microbiota transplantation in refractory patients with IBS-D or IBS-M^60^
HPA axis dysregulation	Epigenetic modifications in the central nucleus of the amygdala (brain region of HPA axis)^61^ contribute to stress-induced VH in rats Chronic stress induces VH and neuronal remodeling in the central nucleus of the amygdala in rats^62^	Lower glucocorticoid receptor mRNA associated with increased pituitary HPA response and severity of overall symptoms and AP in IBS^63^

*VH = visceral hypersensitivity, IBS = irritable bowel syndrome, DSS = dextran sodium sulfate, TNBS = trinitrobenzenesulfonic acid, TRP = transient receptor potential, NGF = nerve growth factor; TRPM8 = TRP melastatin 8; CB1 = cannabinoid receptor 1; CB2 = cannabinoid receptor 2, HPA = hypothalamic-pituitary-adrenal*.

### Preclinical animal models for visceral hypersensitivity

Although it is well recognized that no perfect animal correlate exists due to the complex clinical heterogeneity of IBS, animal studies serve a critical role in gathering basic insights into the biological mechanisms of visceral pain. Preclinical studies have implicated immune dysregulation involving intestinal immune cells and neural-immune interactions^32,33,36,64–67^ in the pathogenesis of visceral pain. In a post-infection (PI)-IBS mouse model of food-induced visceral hypersensitivity, investigators recently demonstrated local IgE production by B cells and plasma cells with subsequent colonic mast cell activation as a mechanism for food-induced visceral hypersensitivity.^32^ Other factors that may contribute to mast cell activation and have been examined in animal models of colonic hypersensitivity include increased expression of toll-like receptor 4 in colonic tissue,^66^, interactions with gut bacteria,^56,68^, bile acid-induced stimulation of mast cell-derived nerve growth factor (NGF),^69^ and stimulation by neuropeptides such as oxytocin via the Ca^2+^-nitric oxide synthase pathway.^70^ Activated mast cells may further trigger visceral pain via upregulation and activation of colonic nociceptors.^30^ In one recent study, Grabauskas et al.^33^ reported that abnormal synthesis and release of prostaglandin E_2_ by colonic mast cells activates the EP2 receptor on neighboring sensory neurons to induce visceral hypersensitivity in rats. Other immune-mediated mechanisms of visceral hypersensitivity that have been described through preclinical models include changes in lamina propria macrocyte phenotype or dendritic cells, altered cytokine signaling, and T-cell activation.^65,71–74^

In addition to aberrant immune responses, there is evidence demonstrating contributions to visceral pain from other related and independent mechanisms. Epithelial barrier impairment may modulate colonic hypersensitivity through mucosal immune activation or by stimulating nociceptive drive. The effects of barrier dysfunction on pain have been well described in preclinical research. For example, increased colonic permeability to sodium fluorescein was associated visceral hypersensitivity in PI-IBS;^32^ inhibition of myosin light chain kinase-mediated intestinal permeability improved visceral hypersensitivity in a *Trichinella spiralis*-infected PI-IBS mouse model;^36^ and decreased expression of tight junction protein, occludin, in colonic epithelial cells resulted in visceral hyperalgesia in rats.^37^ Multiple studies have also demonstrated a role for ion channels including voltage-gated sodium and calcium channels^75–77^, mechanical-gated Piezo proteins,^78^ and transient receptor potential (TRP) channels.^79,80^ In a trinitrobenzene sulfonic acid-induced chronic visceral hypersensitivity mouse model, administration of selective Na_V_1.1-activating toxin enhanced mechanosensory responses to suggest that Na_V_1.1 regulates mechanical hypersensitivity in IBS.^42^ Others have demonstrated that blockade of voltage-gated calcium channels, Ca_V_2.2 and Ca_V_2.3, inhibits colonic nociceptor excitability^41^ and that sensitization of TRP vanilloid 1 (TRPV1) mediates visceral hypersensitivity in rodents.^39,40^

Animal models have further suggested that visceral hypersensitivity may be modulated by the gut microbiome and its mediators (e.g., short-chain fatty acids [SCFA], bile acids, proteases, lipopolysaccharide, histamine) that may act independently or in concert with other aforementioned mechanisms.^31,48,56,57,69,81,82^ Prior studies have shown that antibiotic treatment decreases luminal bacterial counts, enhances bacterial wall adherence, and prevents stress enhanced capsaicin-evoked visceral pain response in mice while sensitivity to colonic distension can be transferred through the fecal microbiota to rodents from patients with IBS.^55,83^ Preclinical studies of SCFA effects have shown than intracolonic acetate enhances sensitivity to colorectal distention^40^ while butyrate-enteric glial cell interactions may regulate NGF and colonic hypersensitivity in rats.^48^ Bacterial neuroactive mediators such as histamine have also been hypothesized to influence visceral hypersensitivity. In a recent study of diet-microbial interactions, germ-free mice colonized with high histamine-producing fecal microbiota exhibited visceral hyperalgesia and mast cell activation.^31^ The gastrointestinal microbiome has generated considerable interest due its potential for shaping visceral sensation through direct effects, interactions with the intestinal immune system and host genetics or diet, and its role in regulating bidirectional communication between the gut and brain.

Other mechanisms underlying visceral hypersensitivity that have been identified through preclinical research include neurotransmitter/peptide-mediated hyperalgesia (e.g., serotonin [5-HT], calcitonin gene-related peptide [CGRP], substance P),^51,69,82,84–87^ altered neuroreceptor signaling (e.g., 5-HT receptors, GABAergic signaling, G-protein-coupled receptors including protease-activated and cannabinoid receptors [reviewed in 69]),^41,85,88–91^ guanylate cyclase C (GC-C) signaling,^92^ stress-induced activation or remodeling of the hypothalamic-pituitary-adrenal (HPA) axis, alteration of descending pain pathways, and sensitization of spinal afferents.^28,61,62,93^

### Pathophysiological mechanisms of visceral hypersensitivity and pain in humans

Evidence gathered from preclinical models of visceral pain and colonic hypersensitivity have guided clinical studies investigating the mechanisms of sensation and pain in patients with IBS, providing opportunities to develop new approaches for diagnosis and treatment. In patients, pain is commonly measured through quantitative traits including sensation thresholds during colonic and/or rectal barostat testing, symptom, or sensation ratings, and neuroimaging-based assessments of brain structure and function. Of the various mechanisms that have been demonstrated in animal studies, many have been examined in humans, with some showing promise as pathways for novel biomarker development. The role of immune dysregulation has been assessed in multiple clinical studies. Two systematic reviews have reported increased mast cell infiltration in the colon and small intestine in patients with IBS compared to healthy controls;^94,95^ however, clinical trials of mast cell stabilizers such as ketotifen in patients with IBS have suggested that mast cell-induced visceral hypersensitivity is largely dependent on the actions of mast cell mediators including histamine on submucosal neurons^35^ rather than overall mast cell density. These findings are consistent with observational studies showing no clear relationship between colonic mucosal immune cells and visceral hypersensitivity in patients with IBS.^96^ In a recent study, detailed inspection of mast cell mediated pathways revealed that injection of food antigens into rectosigmoid mucosa was associated with increased mast cell activation and higher numbers of IgE+ mast cells in closer proximity to nerve fibers in patients with IBS (10 IBS with diarrhea [IBS-D], 1 IBS mixed [IBS-M], 1 IBS unsubtyped [IBS-U]) compared to controls; distance between IgE+ mast cells with nerve fibers was inversely correlated with abdominal pain severity.^32^ Higher levels of systemic proinflammatory cytokines have also been described in IBS patients with pain compared to those without;^97,98^ however, these data are varied and relationships of plasma or colonic cytokine profiles with changes in circulating immune cells including lymphocytes in the context of visceral pain are not fully delineated in patients with IBS.

Epithelial barrier integrity and the “leaky gut” hypothesis has been examined and linked to visceral hypersensitivity and patients with IBS. In one systematic review and meta-analysis, estimated prevalence of barrier dysfunction in IBS ranged from 4% to 62%, was more common in those with IBS-D or PI-IBS, and positively associated with symptoms including pain.^38^ Clinical studies have also provided data to support preclinical observations identifying ion channels as important factors in visceral pain including studies reporting positive associations between colonic transient receptor potential melastatin 8, rectosigmoid TRPV1, or small intestinal TRPV1 or TRPV3 expression levels and abdominal pain in studies of patients with IBS including multiple IBS subytpes,^43–45^ as well as associations of TRPV4 activation and pain scores in IBS-D.^46^ Genetic studies previously demonstrated associations of *SCN5A*-encoded Nav1.5 sodium channel mutations with increased symptoms including pain.^47^ Moreover, clinical trial data have suggested calcium channels as promising therapeutic targets for pain in IBS. Treatment with otilonium bromide, which blocks L-type calcium channel, reduced abdominal pain frequency in a randomized placebo-controlled trial 355 patients with IBS (91 IBS-D, 110 IBS-C, 154 IBS-M);^99^ and treatment with pregabalin, which binds the α2δ subunit on voltage-dependent calcium channels, has been shown to reduce pain and increase sensory thresholds in IBS and especially in patients with IBS-D.^100,101^

In addition to investigating immune cells, epithelial barrier function, and ion channels, many clinical studies have attempted to characterize the role of 5-HT metabolism in IBS pathophysiology. 5-HT is a key gastrointestinal signaling molecule that that acts on both the enteric and central nervous systems (the latter through serotonergic signaling pathways) to modulate gastrointestinal motility, secretion, and nociception. Intestinal enterochromaffin cells produce the vast majority the body’s 5-HT, which is synthesized from tryptophan by tryptophan hydroxylase 1 (the rate-limiting enzyme for 5-HT biosynthesis). 5-HT signaling is ultimately terminated through uptake by serotonin reuptake transporter (SERT) across the plasma membrane where 5-HT is broken down into 5-hydroxy indoleacetic acid (5-HIAA) by monoamine carboxylase.^102^ Admittedly, quantifying effects of 5-HT on visceral hypersensitivity in IBS has proven challenging. Early studies suggested a correlation between mucosal 5-HT and abdominal pain severity.^103^ However, others have been unable to confirm these observations through analysis of tryptophan hydroxylase 1 mRNA levels, SERT transcript levels, or 5-HT in rectosigmoid biopsies of IBS patients with and without rectal hypersensitivity.^104^ In one study, researchers attempted to quantify 5-HT by evaluating both 5-HT and its metabolite, 5-HIAA, in platelet-poor plasma to report lower 5-HIAA levels across all IBS subtypes compared to controls, but no significant differences in 5-HT levels between patients with IBS and controls Although promising, reasons for similarities across subtypes could not be fully explained and laboratory measurement of 5-HIAA levels for IBS remains investigational while relationships with nociception require further study.^105^ Despite promising preclinical data, measuring the effect of 5-HT on abdominal pain in patients with IBS is exquisitely difficult due to multiple factors including variations in sample processing, continued *in vitro* 5-HT metabolism post-sampling, the effects of fasting versus post-prandial states, expression and activity of SERT, transport of 5-HT in the liver/lungs/platelets, 5-HT half-life, and stability, as well as the effects of psychological comorbidities, diet, and consumption of tryptophan-rich foods, and medications.^106^

Direct effects of the intestinal microbiome on pain sensation have also been suggested through clinical studies conducted in patients with IBS. In recent years, researchers have reported associations of relative abundance of colonic mucosal *Prevotella-9* genus with abdominal pain in patients with IBS (28% IBS-C, 38% IBS-D, 24% IBS-M, 10% IBS-U),^58^ correlations of fecal bacterial taxa with sensation and pain,^59^ reduction in pain and discomfort following fecal microbiota transplantation in patients with IBS-D or IBS-M and predominant bloating,^60^ and associations of *Coprococcus* and *Clostridium XIVa* and subcortical regions of the brain involved in pain processing.^107^ However, a clear relationship between microbial structure and pain in IBS has not been fully established and clinical data are often inconsistent or even contradictory.^108–110^ Microbial functions and mediators have also been investigated. While some preclinical studies suggested a role for bile acids in inducing visceral hypersensitivity,^69^ studies in patients have produced conflicting results.^111–113^ Examination of other microbial metabolites and functions have generated data implicating SCFA and microbial metabolism as contributors to visceral hypersensitivity. Vanhoutvin et al. previously demonstrated direct effects of luminal butyrate administration^50^ on sensation thresholds in healthy controls while others have reported associations of fecal microbial enzymes involved in animal carbohydrate metabolism with IBS severity.^114^ One recent study in 29 patients with IBS (11 IBS-C, 10 IBS-D, 8 IBS-M, or IBS-U). and 23 healthy controls further described fecal microbial genes involved in neurotransmitter and SCFA metabolism to be associated with morphometry of the posterior insula, a primary visceral cortex.^115^

Other mechanisms for visceral pain that have been described in preclinical research and further examined through clinical studies of patients with IBS include those related to individual neurotransmitters, growth factors, the endocannabinoid system, and epithelial cell receptor signaling. Clinical research has demonstrated correlations between colonic mucosal substance P and abdominal pain in IBS-D,^52^ negative associations of NGF expression in rectosigmoid biopsies and visceral sensitivity thresholds in IBS-D,^116^ and relief of abdominal pain with linaclotide treatment via GC-C signaling in IBS-C.^92^ Endocannabinoid-like dietary compounds resulted in improved abdominal pain severity in a randomized controlled trial of 54 patients with IBS (27 IBS-D, 10 IBS-C, 17 IBS-M) and 12 healthy controls.^54^ Although convincing clinical trial data supporting the use of cannabinoids for the treatment of visceral pain in IBS are lacking, peripherally restricted agents are recognized as a promising avenue for treatment (reviewed in reference 69). Researchers have also implicated alterations in brain structure, function, and functional connectivity of brain regions related emotional, autonomic, and descending modulatory responses to visceral afferent pain signals^107,117–121^ as well as stress-mediated HPA-axis dysregulation in IBS pathophysiology and visceral pain,^63^ although the specific role of HPA-axis activation in the modulation of abdominal pain has been questioned.^122^ Integrative models have proposed a role for reciprocal interactions between brain networks referred to as the “brain connectome” that co-exist alongside interacting networks with the gut such as the enteric nervous system and the gut microbiota (i.e. the “gut connectome’) in the development, maintenance, and subjective experience of chronic visceral pain (reviewed in^118^ and^123^.In one clinical trial of patients with moderate-to-severe IBS randomized to clinic- vs. home-based cognitive behavioral therapy, responders exhibited changes in white matter integrity and decreased connectivity between brain regions associated with sensorimotor, default mode, salience, and emotional regulation networks; decrease connectivity between the right anterior insula and superior temporal gyrus was further correlated with lower abdominal pain ratings.^124^ It is important to note that despite the focused investigation of distinct mechanisms that mediate visceral hypersensitivity in patients with IBS, many of these mechanisms may be interrelated and/or overlapping and current approaches may offer only partial insights. For example, neuroimaging of innate and elicited brain structure and/or activity be affected by cognitive factors, the nature of the stimulus, or pain expectations.^125,126^ Critical structures such as the brainstem may be relatively inaccessible due to position, size, and physiological noise.^127^ Meanwhile, mechanical stimulation tests such as rectal barostat, may be affected by emotional or neurobehavioral components of pain or yield an incomplete assessment across the gastrointestinal tract including the proximal colon. understanding of human mechanisms and pathways for pain.^128^ Improved understanding of the mechanisms for pain and the best approaches for measuring pain will be crucial for identifying future biomarkers that will pave the way for individualized therapies in IBS.

## OMICS-based biomarkers for abdominal pain in IBS

As a defining feature of the clinical syndrome, developing biological markers that correlate with pain and sensation to provide strong predictive value for diagnosis and outcomes remains essential to shifting the management paradigm from an empiric, symptom-based strategy to one which integrates innovative approaches that will offer a refined assessment of pathophysiology in individual patients. Preclinical and clinical studies have offered mechanistic insights revealing the gastrointestinal microbiome, microbial metabolome, host genetics, intestinal immune system, peripheral and central processing as well as the relationships between these biological networks as critical factors in shaping visceral hypersensitivity. Advancements in our understanding of these mechanisms have helped set the stage for leveraging high throughput omics tools to develop novel biomarkers for pain in IBS.

### Compositional microbiome-based biomarkers for pain in IBS

Preclinical data and human studies show direct links between the gastrointestinal microbiome or microbial byproducts and critical aspects of gastrointestinal physiology including visceral sensation and nociceptive signaling (reviewed in^129^ as well as a role for interactions of the gastrointestinal microbiome with host immune responses, genetics, and diet that further influence clinical phenotype, endophenotype, and treatment response. Studies of community composition and function of the gastrointestinal microbiome through metataxonomic and metagenomic sequencing have become increasingly common in the attempt to uncover microbial mechanisms that underlie IBS pathophysiology, identify candidate microbial IBS biomarkers, and develop novel microbiome-based therapies. Analyses of the gastrointestinal microbiome through the study of fecal specimens as well assessment of mucosal microbial communities in patients with IBS have yielded heterogeneous results. In general, most studies have reported on the associations of microbial structure with clinical phenotype. Common themes include evidence for lower microbial diversity in IBS compared to controls,^130^ decreased temporal stability in IBS, and associations of individual taxonomic groups with IBS and IBS subtypes which have been reviewed by others.^131^ There is less data, however, describing compositional microbial features that appear to be linked to pain or visceral hypersensitivity in IBS (Table 2). Previously, Tap et al.^132^ reported symptom severity in IBS to be associated with fecal microbiome profiles and negatively correlated with enterotypes enriched with *Prevotella* species. However, a recent study by Choo et al.^58^ could not confirm these findings and instead, demonstrated a positive relationship (*r* = 0.36, *p* = 0.0003) between the relative abundance of *Prevotella* genus and *Prevotella_9 copri* from sigmoid biopsy specimens and abdominal pain based on 16S rRNA gene sequencing in a large cohort of patients with IBS, which included patients of multiple IBS subtypes. Others^108^ have examined the fecal microbiome in population-based cohorts to demonstrate a relationship between stool microbiome β-diversity by 16S rRNA gene sequencing, differences in the distribution of *Ruminococcaceae*, *Prevotella*, and *Bacteroides* clusters between those with and without abdominal pain, decreased representation of the *Prevotella*-predominant enterotype in those with pain, and significant differences in abundances of individual taxa between those without and without pain. Notably, *Prevotella* was decreased in those with pain and could predict the absence of abdominal pain. Unfortunately, these findings again, could not be replicated in an independent general population-based cohort^110^ despite the use of similar specimen types and the marker-gene (16S) based approach. In general, comparisons of high throughput sequencing data across studies have been hampered by inconsistencies in sample processing, preparation, sequencing techniques and analytical approaches. While the International Human Microbiome Standards Consortium has recognized that stool sampling requires minimal processing to isolate and maintain microbial DNA and RNA and studies have increasingly targeted the V4 to V3-V4 hypervariable regions for 16S sequencing, substantial phenotypic heterogeneity persists across studies due to factors such as sample size, study design, consideration of patient-specific factors (e.g., diet, geography, clinical subtype), and sequencing approach (16S rRNA vs. random shotgun metagenomic sequencing) which has precluded the identification of a consistent structural bacterial biomarker for pain in IBS.^131,134^ While acknowledging current limitations, metagenomic sequencing continues to improve and will be strengthened by initiatives to standardize approaches to study design, sample collection and processing, analysis methods, and data sharing. It has further offered hope by providing researchers the ability to expand on initial observations through investigation of bacterial gene representation and the assessment of non-bacterial members of the gastrointestinal microbiome. Recent studies have brought to light the potential role of the gastrointestinal virome in IBS pathogenesis which may involve interactions with the gastrointestinal bacteriome;^135,136^ however, direct links between virome composition and visceral hypersensitivity in IBS have not yet been reported. Fungal dysbiosis, on the other hand, has been directly correlated with pain and visceral hypersensitivity in preclinical and clinical research. Botschuijver et al. previously reported differences in fecal mycobiome composition including α-diversity and fungal biomarker species among healthy controls and patients with normo- and hyper-sensitive IBS based on sequencing of fungal internal transcribed spacer regions amplicons. Corresponding animal experiments demonstrated reduction in visceral hypersensitivity with fungicide treatment, restoration of hypersensitivity with transfer of cecal mycobiomes, mast cell histamine release with fungi stimulation, and evidence for fungal recognition via the Dectin-1/Syk pathway with activation of the host immune system as a driver of visceral hypersensitivity.^133^ A subsequent study described reversal of post-stress hypersensitivity with fungicidal miltefosine and reduced TRPV1-induced hypersensitivity in an IBS-like rat model well.^137^ Studies applying high throughput sequencing technologies for evaluation of the gastrointestinal mycobiome, virome, and bacterial genes in modulating visceral sensation in IBS are eagerly awaited. Presently, the available data highlight the importance of considering the full complexity of microbiology ecology through integrated analysis of co-existing bacterial and non-bacterial microbial communities to develop usable microbiome biomarkers for visceral pain. Other important limitations of human microbiome research in IBS include the difficulty of identifying potentially important, but low-abundant taxa, and the incomplete assessment of microbial functions. Moving forward, the clinical relevance of taxonomic profiling and microbial metagenomics may be further advanced through complementary transcriptomics, proteomics, and metabolomics that are designed to measure microbial functions and microbe–host interactions important for mediating nociception in IBS.

**Table 2.** Potential microbiome-based biomarkers for visceral pain in humans.

Microbiome Variable	Sample Type	Source	Evidence	Study (reference)
Beta diversity	Stool	Population-based cohort	β-diversity correlated with multiple pain indices	Hadizadeh et al.^108^
Beta diversity	Stool	Population-based cohort	No association of β-diversity with abdominal pain	Frost et al.^110^
Microbial richness	Stool	Patients with IBS	Negatively associated with symptom severity	Tap et al.^132^
Enterotypes enriched with *Prevotella*	Stool	Patients with IBS	Negatively associated with symptom severity or pain	Tap et al.^108;132^
*Prevotella_9 copri*	Mucosa	Patients with IBS	Relative abundance positively associated with abdominal pain	Choo et al.^58^
*Blautia, Streptococcus, Lactobacillus*	Stool	Population-based cohort	Increased in those with pain	Hadizadeh et al.^108^
Fungal mycobiome	Stool	Healthy volunteers; Normo- and hypersensitive IBS	Altered community composition in hypersensitive IBS	Botschuijver et al.^133^

### Targeted and untargeted metabolomics reveal mediators of pain and sensation in IBS

Metabolomics data serve as a rich source of information on the small molecule mediators, including microbial metabolites, that influence visceral hypersensitivity and could be used as novel biomarkers for pain in IBS (Table 3). Microbially derived metabolites including bile acids, SCFA, serotonin, and similar metabolites such as monoamine tryptamine are of considerable interest due to their role in gastrointestinal physiology and their potential effects on IBS pathogenesis including mechanisms related to pain. Recent studies^143–145^ have examined targeted and untargeted metabolite profiles from various biospecimens (e.g., stool, urine) to report differences between patients with IBS compared to controls and between IBS subtypes as well as a correlation between gastrointestinal metabolites and physiological functions (e.g., intestinal fluid secretion) or symptoms (e.g., stool type). Measurement of fecal bile acids has been validated a biomarker for bowel functions and clinical phenotype in IBS; however, their role as biomarkers for visceral hypersensitivity appear more limited.^146^ Targeted assessment of SCFA effects on visceral sensation in preclinical studies have provided evidence for increased colorectal hypersensitivity with luminal administration of SCFA including butyrate.^48,49^ However, these data have not been verified in clinical studies, including studies that have reported decreased pain scores according to the barostat protocol with daily administration of butyrate enemas in healthy volunteers.^50^ Overall, validating luminal SCFA as individual biomarkers for pain or treatment responsiveness in IBS has proven challenging^31^ due to the dynamic nature (e.g. rapid uptake, utilization for cross-feeding) and role of SCFA within the gut. SCFA may also be linked to sensation through unmeasured mechanisms including the metabolic pathways for SCFA production and downstream SCFA effects on host physiology. For these reasons, future investigations of SCFA profiles will benefit from integrated analysis incorporating multi-omics tools that will offer a fuller picture of the role for SCFA as candidate IBS biomarkers and mediators of pain.

**Table 3.** Potential metabolite biomarkers for visceral or abdominal pain in humans.

Metabolite	Sample Type or Site action	Study Population	Evidence	Reference
Butyrate	Colon	Healthy volunteers	Colonic administration of butyrate decreased pain, urge and discomfort assessed by rectal barostat	Vanhoutvin et al.^50^
Histamine	Urine	Patients with IBS	Improved abdominal pain in patients with IBS after restriction of fermentable carbohydrates associated with lower concentrations of urinary histamine	McIntosh et al.^138^
Histamine	Rectal biopsy specimens	Healthy volunteers	TRPV1, TRPA1 and TRPV4 responses of submucosal neurons potentiated by histamine	Wouters et al.^35;^ Balemans et al.^139^
Tryptophan	Stool	Patients with IBS	Tryptophan concentration higher in IBS with symptom exacerbation versus IBS without symptom exacerbation	Tanaka et al.^140^
Volatile organic compounds (VOCs)	Breath	Patients with IBS	16 VOCs correlated with symptom scores including abdominal pain and discomfort	Baranska et al.^141^
Metabolic profile	Stool	Patients with IBS	Metabolite clusters significantly correlated with abdominal pain and discomfort	Zhu et al.^142^

Data derived from metabolomics studies have also identified other important mediators including histamine, which may be produced through mast cell degranulation and as a bacterial metabolite^31^ as well as metabolites affecting the serotonin biosynthetic pathway. In a prior study conducted in patients of any IBS subtype, metabolomics assessments demonstrated decreased urinary histamine following a low fermentable oligosaccharide, disaccharide, monosaccharide, and polyol diet, which was associated with reduced symptom severity as well as increased bacterial richness.^138^ Concentrations of histamine and its metabolites have also been associated with TRP channel sensitization, and therefore recognized as important candidate biomarkers for visceral pain perception in studies of patients with IBS (18 IBS-D, 7 IBS-C, 4 IBS-M, 10 IBS-U).^139^ Another recent study analyzed fecal metabolomics data in patients with IBS-D to observe increased tryptophan concentrations in patients with symptom exacerbation.^140^ Others have studied nontargeted metabolite profiling including volatile organic compounds in breath and fecal metabolite profiles as potential tools, and therefore biomarkers, for identifying clinical symptoms and outcomes.^141,147^ Despite promising signals, rigorous assessment with standardization of optimal specimen acquisition and technical methods^148^ as well as validation in independent cohorts will be required before such metabolomic panels can be adapted as biomarkers for evaluating pain or hypersensitivity in clinical practice.

### Genetic and epigenetic biomarkers for visceral hypersensitivity in IBS

Preclinical and clinical studies investigating genetic determinants of pain and visceral sensation have focused largely on identifying genetic variations associated with immune dysregulation, barrier function, neurotransmitter biosynthesis and metabolism, cannabinoid receptors, ion channel dysfunction, and G-protein coupled receptor expression alongside other mechanisms.^27,149^ The increasing availability of large-scale population-based biobanks have facilitated advancements in genomic research in IBS^150^ that may accelerate our understanding of genetic risk determinants in IBS. In one genome-wide association study of 53,400 people with IBS, six genes (*NCAM1, CADM2, PHF2/FAM120A, DOCK9, CKAP2/TPTE2P3* and *BAG6*) were found to be associated with IBS susceptibility including four genes linked to mood, anxiety, or expressed in the nervous system. Although genetic mechanisms of visceral hypersensitivity were not specifically examined, these findings provide evidence that genes involving neuronal pathways may play a major part in shaping abnormal brain-gut interactions in IBS.^151^ Recently, investigators identified new candidate genes involved in degradation of epidermal growth factor (*SNX13*) and histone methyl-lysine binding protein (*L3MBTL4*) that were associated pain severity and frequency in IBS.^152^ The reader is referred to a prior paper that provides an in-depth review on the genetics of visceral sensation in IBS.^27^ Focused genomic studies have also implicated SERT gene (*SLC6A4*) polymorphisms in visceral hypersensitivity in IBS. However, a systematic review and meta-analysis examining the relationships between *SLC6A4* polymorphisms and IBS revealed no significant overall association. Instead, associations of mutations in the polymorphic region (5-HTTLPR) of *SLC6A4* and IBS-C were observed only in the East Asian population to suggest that assessment of genetic variations may be more useful for pharmacogenomics and in predicting responses to drug therapies^153^ than as biomarkers for IBS. Although large-scale studies have hinted at genetic heritability of IBS and visceral hypersensitivity,^151^ functional contributions of genes to pain in IBS may be modest, determined by epigenetic modification and gene expression, and influenced or even overshadowed by environmental factors.

Epigenetic modulation of gene expression has been proposed as another important factor in shaping gene-environment interactions that influence mechanisms of visceral pain in IBS and thus, hold promise as novel diagnostic and prognostic biomarkers. Epigenetic changes that mediate visceral hypersensitivity can include histone tail acetylation or modification, differential microRNA (miRNA) expression, and DNA methylation.^154^ Studies of epigenetic contributions to visceral pain in IBS have examined epigenetic modulation of stress-associated activation of the HPA-axis, SERT, and TRPV1. Epigenetic marks defined by decreases in histone acetylation at the corticotrophin-releasing hormone (CRH)- and glucocorticoid-receptors were recently shown to contribute to stress-mediated visceral hypersensitivity in rats.^61^ Others have published evidence to suggest a role for miRNA in regulating gene expression of SERT and TRPV1. Liao et al.^86^ previously demonstrated upregulation of miR-24 in patients with IBS and SERT as a potential gene target; treatment with miR-24 inhibitor increased colonic pain and nociceptive thresholds in mice. In another study, colonic miR-199a/b correlated with pain and TRPV1 expression in patients with IBS-D.^155^ In the future, it is possible that integrated studies examining genetic variants, epigenetic markers, gene expression and gene-environment interactions through complementary analyses of the microbiome and metabolome will offer refined genetic biomarkers targeting diagnosis and drug development for visceral hypersensitivity in IBS.

### Transcriptomics for identifying biomarkers for visceral hypersensitivity

High throughput transcriptomics technologies including microarray and RNA-sequencing (RNA-seq) have emerged as valuable tools for unraveling the complexities of the functional genome and pathobiology of visceral hypersensitivity in IBS. The role of transcriptomics biomarkers for IBS and more specifically, for abdominal pain, has been explored in multiple studies. In a pilot cohort of 9 patients with IBS-D and 9 controls, RNA-seq of rectosigmoid biopsy specimens demonstrated transcriptome changes involving neurotransmitters, ion channels, immune function and cytokines, barrier function, as well as cell adhesion in patients with IBS.^156^ Microarray analysis of rectal mucosal specimens and bioinformatics approaches have recently implicated genes including *GRPR*, neuropeptide FF (*NPFF*), and *TRPA1* to be associated with abdominal pain in IBS-D.^149^ Gene expression profiling of colonic biopsy specimens with microarray analysis has also demonstrated pathways involved in 5-HT metabolism to be differentially expressed between IBS and controls, suggesting transcriptional patterns of serotonergic gene expression including TPH1 as potential biomarkers for IBS.^157^

Recently, transcriptomics has been used for more focused studies of visceral hypersensitivity. Using chronic water avoidance stress (WAS) rodent model, Wiley et al. demonstrated changes in gene expression within colonic epithelial cells of genes involved in inflammatory and immune responses including cytokine- and chemokine-receptor, adherens, and tight junction genes to suggest alterations in the expression of these genes as prospective biomarkers for impaired permeability and visceral hypersensitivity in IBS.^158^ Transcriptomics has also enabled researchers to investigate mechanisms of stress-induced visceral hyperalgesia related to CGRP, mast cells, and CRH to identify novel gene targets for the diagnosis and treatment of IBS. In a preclinical WAS model, gene expression of colonic mast cells was analyzed by RNA-seq to demonstrate increased expression of genes related to response to CRH, transcription regulation, mast cell activation, inflammation, and proliferation. Findings suggested that CGRP may regulate mast cell activation and mediate stress-induced hypersensitivity through CRH in the brain-gut axis.^87^ In patients with both IBS-D and IBS-C, transcriptomics analysis of small intestinal biopsies have also demonstrated correlations of abdominal pain and rectal sensation thresholds with changes in duodenal TRPV1 and TRPV3 expression. Results indicated that visceral hypersensitivity in IBS may be mediated by altered intestinal chemosensitivity and that intestinal transcriptomics may be useful for evaluating visceral pain^45^ Others have applied RNA-seq to study bacterial transcriptomes and gene expression in peripheral blood. These studies have detected differences in bacterial transcriptomes related to butyrate production and neuroendocrine hormones between IBS-D and health^140^ as well as relationships between expression of inflammatory genes in peripheral blood mononuclear cells and brain regions within the salience network, which is involved in modulating cognitive processing including attention to pain;^159^ however, direct associations with abdominal pain and colonic sensation were not reported. Although encouraging, findings from recent transcriptomics studies should be interpreted cautiously considering factors such as the relative inaccessibility of the intestine with limited sample collection via traditional biopsy methods and cell-to-cell heterogeneity that may be masked by bulk transcriptomic profiling. While no single transcriptomics-biomarkers have been fully validated for clinical use, transcriptomics including next-generation single-cell RNA-seq represent an important tool for disentangling the genetic mechanisms of abdominal pain in IBS and represents a crucial layer of multi-omics discovery research (Table 4).

**Table 4.** Potential biomarkers for abdominal pain in human transcriptomic studies.

RNA Transcript	Sample Type	Source	Evidence	Reference
mRNA	Mucosa (rectosigmoid)	Patients with IBS	*GRPR*, *NPFF* and *TRPA1* genes as potential biomarkers for abdominal pain in IBS	Lin et al.^149^
mRNA	Mucosa (duodenal)	Healthy Volunteers; Patients with IBS	Positive correlation between *TRPV1* and *TRPV3* expression expressions and abdominal pain; Inverse correlation between *TRPV1* and *TRPV3* expressions and threshold for first rectal sensation and pain	Grover et al.^45^
mRNA	peripheral blood mononuclear cells	Patients with IBS	Pro-inflammatory genes, *IL6* and *APOL2*, positively correlated with salience network regions implicated in visceral sensitivity	Gupta et al.^159^

### Proteomics for advancing biomarker discovery for abdominal pain and IBS

Modern proteomics technologies have shown great promise for addressing key bottlenecks in biomarker development for abdominal pain and IBS, offering a unique advantage of more closely representing endophenotype and clinical phenotype than genomics or transcriptomics-based analyses. Untargeted proteomics may be used as a strategy for high throughput biomarker screening that can be further validated by targeted proteomics approaches.^24^ In a recent study of patients with PI-IBS, fecal metaproteomic specimens assays were used to identify host proteases associated with high proteolytic activity in feces, which has been linked to barrier dysfunction and visceral hypersensitivity. Complementary analyses of the fecal microbiome further revealed that specific members of the intestinal microbiome could suppress proteolytic activity through microbial β-glucoronidase.^160^ Metaproteomics has also been used to study colonic mucus samples from patients with IBS-D to demonstrate high rates of *Brachyspira* colonization through identification of bacterial peptides/protein, as well as associated changes in the host mucus proteome involving inflammatory mediators and membrane remodeling proteins. Researchers demonstrated that spirochetosis was not associated with symptom severity or rectal sensitivity, but was correlated with other clinical and quantitative traits including transit, bowel symptoms, mucosal inflammation, and mast cell activation that have been linked pain in IBS.^161^ While still in the early stages of application, proteomics-based tools may represent a strategy to overcome technical and conceptual challenges in biomarker discover. Similar to other omics strategies, metaproteomics may benefit from complementary single-cell approaches to account for cell-specific heterogeneity.

### Brain connectome in IBS and abdominal pain

Other omics approaches that have considered in the effort to develop reliable biomarkers capable of measuring pain and treatment response in IBS include “connectomics” which utilizes noninvasive neuroimaging to study brain neural elements and networks. A prospective study of 84 IBS patients from a randomized controlled trial of cognitive behavioral therapy (CBT) examined brain resting state functional connectivity and microstructure to observe reduced functional connectivity between brain regions associated with sensorimotor, default mode, salience, and emotion regulation networks as well as changes in white matter in patients exhibiting treatment responses, measured by IBS symptom severity including features such as pain. However, prediction to CBT response was more accurately predicted by baseline microbiota than by neuroimaging data to suggest microbial composition as a more promising biomarker. In another study, Icenhour et al.^117^ compared resting state functional connectivity between normosensitive and hypersensitive patients with IBS to report that visceral sensitivity correlated with changes in functional connectivity of networks involved in salience, sensory processing and interoception. In the future, big neuroimaging data may offer promising brain-based biomarkers for identifying IBS endophenotypes or predicting responses in symptoms such pain to pharmacological treatments and other therapies.^120^

### Moving together through multi-omics for biomarker discovery and validation

**Figure 1.**
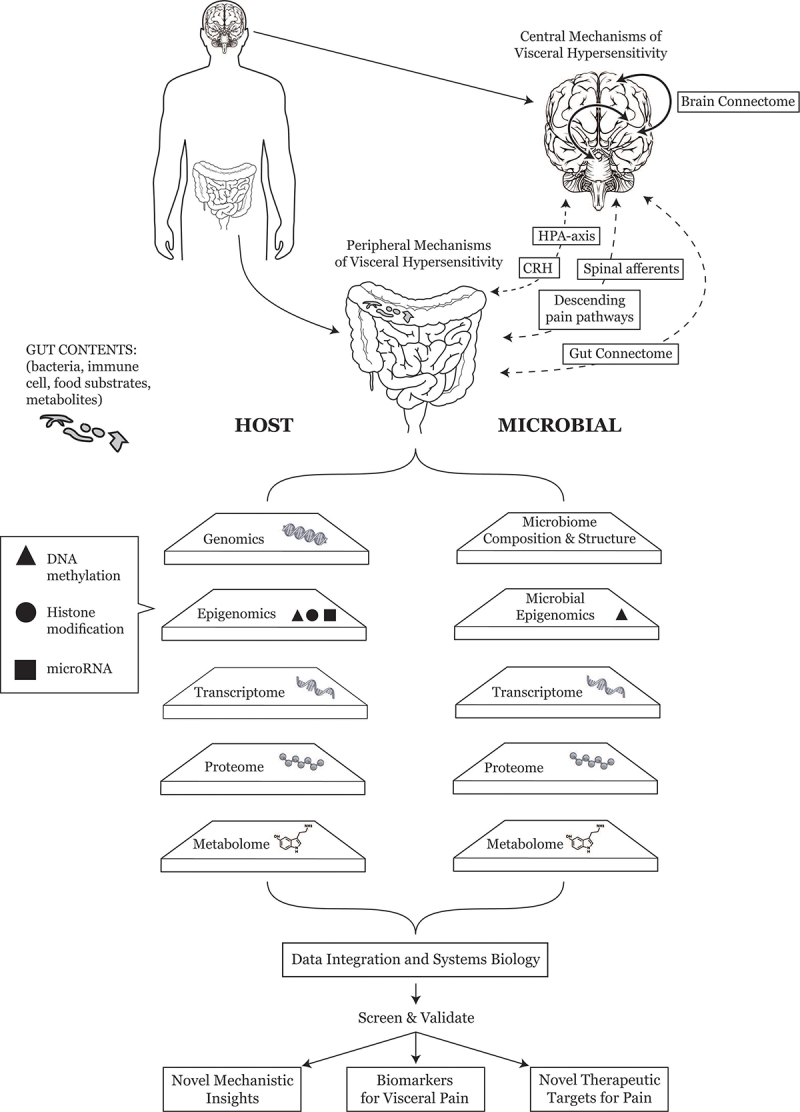
Conceptual model of multi-omics approaches for biomarker discovery in abdominal pain and irritable bowel syndrome (CRH = corticotrophin-releasing hormone; HPA = hypothalamic-pituitary-adrenal).

Advancements in metataxonomics, metagenomics, metabolomics, genomics, epigenomics, transcriptomics, proteomics, and even connectomics have made it apparent that integrated analyses of complementary omics data will be crucial for understanding the complex biological networks that underlie the interactions between the gastrointestinal microbiome and human physiology in a clinically meaningful manner. Through multi-omics discovery science, researchers have begun to delineate novel biological pathways and dynamic microbe–host interactions that may drive IBS pathophysiology; these insights may further facilitate the identification of candidate biomarkers with diagnostic, prognostic, or therapeutic value (Figure 1). In a longitudinal multi-omics study of the gastrointestinal microbiome, metabolome, host epigenome, and transcriptome, investigators recently identified host-microbial pathways associated with purine metabolism as a novel feature in IBS that could be linked to symptom pathophysiology through reduced epithelial energy and mucosal repair.^143^ Another recent study used multi-omics approaches to examine fecal proteolytic activity, which has been associated with increased symptom severity and higher intestinal permeability, in patients with PI-IBS to observe increased abundance of human serine proteases using metaproteomics.^160^ Transcriptomic analysis of the colonic mucosa revealed that differences in host proteases were not explained by mucosal expression of proteases of protease inhibitors. Instead, complementary metagenomics and metabolomics analyses determined intestinal proteolytic activity to be regulated by the commensal microbiome including *Alistipes putredinis* and gut microbial β-glucuronidase-mediated production of unconjugated bilirubin, suggesting a novel host-microbial interaction that could represent a future biomarker or therapeutic target for PI-IBS and more specifically, impaired permeability or visceral hypersensitivity. Others have employed 16S rRNA sequencing, bacterial metatranscriptomics, and metabolomics to examine contributions of microbial structure and function to clinical symptoms and phenotype among healthy controls and IBS-D patients with and without symptom exacerbation to identify changes in fecal omics profiles associated with symptom exacerbation including decreased *Bifidobacterium longum* as well as differences SCFA and neurometabolites and bacterial gene transcripts related to butyrate production and neuroendocrine hormones, highlighting the utility of multi-omics profiling of the colonic microenvironment as an indicator of IBS symptom phenotype.^140^ Multi-omics studies have also been performed to generate novel insights into the interactions between the gastrointestinal virome, bacteriome, host transcriptome, and metabolome in healthy controls and patients with IBS. In a subset of individuals from a larger multi-omics study, investigators recently reported temporal stability of the intestinal virome, associations between the virome and host colonic genes linked to immune responses and epithelial barrier function, associations of bacteriophage composition with clinical phenotype, as well as correlations between viral contigs and bacterial species, functions, and the primary bile acid chenodeoxycholate.^136^ These findings, while preliminary, provide a comprehensive assessment of the broader microbial ecosystem and the interactive contributions of bacterial and non-bacterial microbes to IBS pathophysiology through combined omics analyses that could guide the future selection of important diagnostic and/or predictive microbial biomarkers. New machine-learning approaches for integration of paired 16S rRNA sequencing and host transcriptome data from colonic mucosal specimens have further been developed to identify host gene–microbe interactions that are specific to IBS and common to various gut disorders including IBS, colorectal cancer, and inflammatory bowel disease.^162^ Priya et al. observed overlapping host gene pathways to exhibit disease-specific correlations with microbial taxa. For example, the RAC1 pathway, which is involved in immune response and intestinal mucosal repair, was correlated with *Bacteroides massiliensis*, *Bifidobacterium*, and *Odoribacter* in IBS. Other IBS-specific associations included associations of *Prevotella* with pathways for sumoylation (DNA damage response and repair proteins), *B. massiliensis* with arachidonic acid metabolism (epithelial homeostasis), Peptostreptococcaceae with *HAS2* (colonic epithelium protection), and *Streptococcus* with *DPEP2* (macrophage inflammatory response). Systems biology approaches have also been described through combined analyses of brain connectivity data with other omics datasets in patients with IBS. In a cross-sectional analysis of 102 women (36 IBS-C, 27 IBS-D, 39 healthy controls) Sarnoff et al. utilized a multi-omics data integration analysis method for biomarker discovery (DIABLO) to integrate fecal microbiome, metabolome, resting-state connectome, and clinical data to identify brain-gut-microbiota signatures that differentiated IBS subtypes from healthy controls.^163^ Bloating and visceral sensitivity were negatively associated beneficial taxa such as *Blautia obeum* and with brain connectivity involving the orbitofrontal cortex. Although visceral perception was not directly assessed, these findings hold significant potential as they may pave the way for future research in biomarker development through the study of multidimensional omics data.

Studies such as these offer invaluable information on the host–microbe interactions that mark and shape IBS pathophysiology, while outlining powerful strategies for data integration. Increasingly, researchers have begun embracing integrative multi-omics as a powerful and systematic approach to studying the many layers of molecular and biological events that link genotype to clinical symptoms, with immense potential for identifying reliable candidate biomarkers for visceral pain in IBS. Indeed, some have begun combining multi-omics with digital therapeutics to demonstrate that changes in IBS symptom severity can be modeled through a mixture of genomic and microbiome predictors, suggesting that prognostic multi-omics biomarkers for IBS symptomology are already on the horizon.^164^

## Concluding remarks

High throughput single-omics technologies have fueled significant progress in our understanding of the biological mechanisms of visceral pain and provided new opportunities for biomarker discovery research in IBS as well as other disorders of gut–brain interaction and diseases of the digestive tract for which mechanisms of pain are not yet fully defined. Individually, omics technologies have revealed key mechanisms within microbial communities and host molecular pathways that may underlie the development of visceral hypersensitivity in IBS. Microbiome studies have detected structural and functional features including individual taxa such as *Prevotella* or microbial β-glucoronidase as well as non-bacterial microbes; metabolomics have identified critical mediators of host physiology; genetic or epigenetic and transcriptomic research have shed light on important genetic differences involving ion channels, serotonin metabolism, immune activation, and HPA-axis dysregulation; proteomics has provided opportunities to infer functional microbe–host interactions within the gut; while connectomics has enabled researchers to employ new technical and conceptual approaches to studying brain signatures as biomarkers for visceral pain perception. In parallel, advanced computational tools and statistical methods designed to combine large-scale data from different omics approaches are continuously emerging, allowing researchers to screen for biomarkers that could be used for IBS and visceral hypersensitivity through simultaneous investigation of multiple functional layers within overlapping biological systems that include contributions from both the host and the resident microbiome. As major advancements are underway, there remain important limitations of omics-based tools that will need to be addressed before these approaches can be used in the clinical setting. Analysis of compositional microbiome-based biomarkers for pain in IBS is challenged by significant heterogeneity across studies and an incomplete understanding of microbiota–host interactions. Metabolomics research holds significant promise, but inherent technical limitations of metabolite quantification remain and clinical validation is required to fully interpret the pathophysiological consequences of targeted and untargeted metabolomics data. Genetic contributions to pain in IBS may be largely shaped by epigenetics, gene expression, and environmental variables. Standardization and refinement of neuroimaging techniques including the incorporation of multimodal imaging modalities will be needed to more fully understand the impact of the central nervous system on bidirectional brain–gut interactions. Furthermore, bulk omics-based approaches aimed at conducting high throughput analyses of biological molecules and samples may mask or fail to capture microbe- or cell-specific variations in qualitative and quantitative functions.^165^ For these reasons, further research including longitudinal work, large-scale studies, integration of novel single-cell technologies, and incorporation of clinical meta-data is necessary to validate the findings and deep biological insights that are being gathered through innovative multi-omics science. Yet, with the arrival of the omics era, the biomedical research community is now positioned to investigate dynamic microbe-host interactions and that can be leveraged to accelerate precision medicine. Together, multi-omics approaches hold immense potential for driving novel biomarker prediction, development, and implementation to enhance the diagnosis and treatment of visceral pain in IBS.

## Data Availability

Data sharing is not applicable to this article as no new data were created or analyzed in this study.

## Abbreviations

CBT = cognitive behavioral therapy; CGRP = calcitonin gene-related peptide; CRH = corticotrophin-releasing hormone; DGBI = disorders of gut brain interaction; EP2 = prostaglandin E2 receptor; GABA = gamma-aminobutyric acid; GC-C= guanylate cyclase C; GRPR = Gastrin releasing peptide receptor; HPA = hypothalamic-pituitary-adrenal; IBS = irritable bowel syndrome; IBS-D = IBS with diarrhea; miRNA = microRNA; NGF = nerve growth factor; NPFF = Neuropeptide FF; PI = post-infection; SERT = serotonin transporter; RNA-seq = RNA-sequencing; SCFA = short-chain fatty acids; TRP = transient receptor potential; TRPA1 = TRP cation channel subfamily A member 1; TRPV1 = TRP vanilloid 1; TRPV3 = TRP vanilloid 3; TRPV4 = TRP vanilloid 4; WAS = water avoidance stress; 5
